# Postscript

**DOI:** 10.1007/978-3-030-55400-2_7

**Published:** 2020-10-17

**Authors:** Joseph E. Stiglitz

**Affiliations:** 1 Office of the President of the Republic of Finland, Helsinki, Finland; 2 Faculty of Law, University of Helsinki, Helsinki, Finland; Columbia University, New York, NY USA

## Abstract

The Covid-19 epidemic has unveiled inequalities and instabilities in national and global economic and social systems of many countries, even rich and powerful ones such as the United States. The major economic and social crises that have followed signal that it is now time to reflect on their causes, namely poorly managed globalization and lack of global cooperation before the pandemic. Looking to the ILO as a model, the article calls for an imperative to construct a post-pandemic world marked by more global cooperation, with better social governance.

The Covid-19 epidemic has exposed many of the deficiencies in national and global economic and social systems. For all its wealth, the seemingly richest and most powerful country, the United States, became the global leader in infection rates, deaths, and an increased unemployment rate. The riots that broke out across the country in May, 2020, signified a country greatly divided. Money can buy a lot of things, but evidently it alone can’t buy health or social harmony.

The reasons for the dismal failure of the US are manifold: Unfettered capitalism led to unbridled inequalities and unmatched instabilities, exemplified by the 2008 financial crisis in which ordinary citizens who lost their homes and jobs bore the brunt of the costs; corporations and banks have been allowed to exploit their customers, their workers, and the planet that we share;^1^ but among the most important reasons for the US failures is the inadequacy of its systems of social protection and the denigration of the rights of workers.

The connections are, unfortunately, all too clear.^2^ The US is the only advanced country that doesn’t recognize the right to health care as a basic human right. And while President Obama tried to extend access to health care for all, President Trump and his party have worked hard to reduce it, so that 3 years after taking office, some two million more Americans are without health insurance. The minimum wage in the United States is lower than it was 60 years ago, adjusted for inflation, and that means working fulltime doesn’t generate a livable income. Large fractions of Americans live paycheck to paycheck. On top of that, America is one of the few advanced countries not to mandate paid sick leave. The combination—living paycheck to paycheck and no paid sick leave—is lethal, because it means that employees infected with Covid-19 go to work when they possibly can. They have to, if they are going to feed their families and pay their bills.

Congress recognized the problem, and passed a law requiring paid sick leave, but just for workers sick with Covid-19. Then, under pressure from America’s biggest and richest companies, they exempted employers with more than 500 employees—precisely the employers who could most easily pay for it. Thus, almost half of all workers were exempted. This story tells a lot about the short-sightedness and the selfishness of capitalism American-style—even the corporations lost. For instance, when meat packing companies, which failed to provide masks and protective gear for their workers, became hot spots of the disease, the plants had to shut down. This cost the companies far more than they would have saved by not providing paid sick leave. With such pervasive myopia, it explains why government must play a role.

The corporate decisions might have been different if workers had had a say on company boards. The workers might have been able to explain that not providing health insurance or paid sick leave was penny wise but pound foolish, because a healthy labor force is a more productive labor force. Governance matters. Who makes the decisions affects what decisions are made. And good economic governance has to pay attention to social consequences.

Indeed, the objective of our economic system should not be to just increase GDP—or corporate profits. A well-functioning economic engine should be designed to raise the living standards—broadly understood—of all citizens. GDP, it is now recognized, is a poor measure of societal well-being, or even economic performance more narrowly defined.^3^

It used to be that workers’ perspectives could be presented strongly by unions. Here in New York City, the butchers’ union made it clear during the pandemic that their workers weren’t going to work unless employers provided masks. But in the US and many other countries, unions have been weakened to the point where they are unable to adequately advocate on behalf of workers.

## Globalization

Underlying the problems that Covid-19 has exposed so dramatically is the weakening of the power of workers—their bargaining power vis-a-vis corporations in the marketplace, and their political power, as corporate money has held increasing sway in America’s money-driven politics. (I should emphasize: I write about America both because it is the country I know best, but also because it has become the paradigm for what happens with unbridled capitalism. Too many countries, especially before the 2008 crisis, looked to the US as a model, to their detriment. Those countries that have most closely emulated the American model have the highest levels of inequality and the worst performance across a wide range of social dimensions.^4^)

There are, in turn, multiple intertwined reasons for this weakening of workers’ political and economic power. The weakening of unions has had political consequences; imbalances in wealth and economic power inevitably translate into imbalances of political power, and the consequence is legislation that makes workers’ collective action more difficult—more difficult to unionize and to achieve gains at the bargaining table.

Poorly managed globalization is one of the important reasons for the change in the power of workers. As emerging markets and less developed countries became more integrated into the global economy, workers in advanced countries were pitted against workers from the developing world. Standard theory predicted that this would lower wages of workers in the advanced countries, especially if they were unskilled. Indeed, this process would continue until wages (adjusted for skills) were the same everywhere—that’s the ideal of a well-functioning market. Of course, the advocates of unfettered globalization never “advertised” that this would be the outcome. Rather, they told another story, of globalization making countries richer, of benefits trickling down to everyone in society. There was never any theory or evidence behind these arguments, they were just shiny lures—and now four decades have shown to be true what serious economists had predicted all along: stagnant incomes for large fractions of workers and a hollowing out of the middle class.^5^

Of course, globalization could have been managed in other ways, but it was managed by and for corporate interests. These interests, for instance, succeeded in getting stronger property rights protections outside the United States than corporations had within the US. The companies could then threaten to move abroad unless their workers gave concessions on wages and working conditions.

Again, the pandemic has illustrated the short-sightedness of this unbalanced globalization, where the United States was incapable of quickly producing the masks, the protective gear, the ventilators, and the tests that the country required. A focus on short-term profits had made the economy far less resilient, far less able to respond.

## Global Cooperation

Meanwhile, the pandemic has illustrated both the negative and positive sides of global cooperation. The pandemic and its economic consequences will not be contained until it is contained everywhere, and unless there is a global economic recovery. Pandemics and global environmental issues, like climate change, are arenas where global cooperation is absolutely essential. Fortunately, the world has, over the past hundred years, created international organizations to manage global cooperation. In this arena the lead organization is the World Health Organization, but the multilateral financial institutions have played an important role in providing finance for poor countries to strengthen their health care systems.

At the same time, scientists around the world are cooperating in the attempt to quickly discover a vaccine, develop better tests, and find therapies that are effective against the disease.

That’s the positive side. On the negative side, the American president pulled out of WHO, and has undertaken policies to ensure that the US has first access to any vaccine—rather than that the vaccine goes to where it is most critical, e.g. to health care workers. While other countries have committed themselves to a Covid-19 patent pool—to ensure that the knowledge will be available to all, with appropriate licensing fees—so that the scourge will be eliminated as quickly as possible, the US has focused on strengthening intellectual property claims.

Almost 20 years ago, I served on the World Commission on the Social Dimensions of Globalization established by the ILO. This 101-year-old organization is distinctive in bringing together governments, workers, and businesses to address the common problems we face, and working to achieve solutions that are in the common interest. Earlier discussions of globalization had largely forgotten its social dimensions. As I pointed out earlier, unfettered globalization—or more accurately, globalization managed for the benefits of large corporations—can result in a race to the bottom, with wages and working condition deteriorating for many in the advanced countries. The environment, too, will be a victim of this kind of globalization. We didn’t fully see that even the economy can be a victim, as became apparent during the global financial crisis a short five years after the issuance of our report.

One area in which we had a healthy discussion was intellectual property, where the particular concern was access to medicines, precisely the issue raised by the patent pool. It should be clear that most of the key advances in this area rest on foundations of publicly funded research. Moreover, there are better ways to conduct testing, at lower costs, and with fewer conflicts of interest, than by allowing the very companies that stand to profit from a drug to be responsible for its testing. The drug companies do perform a role in bringing the drugs to market, though the way they do this often results in exorbitant prices and actually impedes innovation.

Our Commission concluded that the intellectual property regime established as part of the Uruguay Round that had created the WTO, the so-called Trade Related Intellectual Property system (TRIPS), was badly flawed. We needed “TRIPS-minus,” that is, a system of intellectual property rights that also guaranteed the rights of access, especially for life-saving drugs. The drug companies, not surprisingly, supported by the US and some other governments, have, to the contrary, succeeded in creating in the subsequent years a “TRIPS-plus” regime, one that further restricts access to medicines, a regime that may prove particularly costly in this pandemic.

## Concluding Comments

Crises provide moments for reflection. The 2020 pandemic is a major crisis—a health crisis precipitating a major economic and social crisis. It is a global phenomenon, requiring a global response. It is a crisis that highlights the inequalities in our society. The virus is not an equal opportunity infector—it goes after people in poor health; and in societies like the US, marked by high levels of health and income inequalities, there are many in poor health.

Had there been more global cooperation before the pandemic—cooperation in which *all* voices were heard, not just the corporations’—we might have built a world marked by less inequality. We might have constructed more resilient economies that were better able to cope with the pandemic and with its economic consequences.

Now, the imperative is to construct a post-pandemic world marked by more global cooperation, with better social governance, with the voices of all stakeholders being heard. The ILO provides a model of what can be done.

## Footnotes

1 I expand on many of the themes raised in this post script in my recent book, Stiglitz (paperback edition, with new introduction, 2020).

2 For a further discussion of these issues, see Stiglitz (2020).

3 See Stiglitz et al. (2010, 2018).

4 Overall, countries with greater inequality also have poorer economic performance, more narrowly defined. See, e.g., Stiglitz (2012).

5 For a more extensive discussion of the points raised in this paragraph, see Stiglitz (2017).
